# Summary of the Standards, Options and Recommendations for the management of patients with carcinoma of unknown primary site (2002)

**DOI:** 10.1038/sj.bjc.6601085

**Published:** 2003-08-15

**Authors:** R Bugat, A Bataillard, T Lesimple, J J Voigt, S Culine, A Lortholary, Y Merrouche, G Ganem, M C Kaminsky, S Negrier, M Perol, C Laforêt, P Bedossa, G Bertrand, J M Coindre, K Fizazi

**Affiliations:** 1 Institut Claudius Regaud, Toulouse, France; 2 FNCLCC, Paris, France; 3 Centre Eugène Marquis, Rennes, France; 4 Centre Val d’Aurelle, Montpellier, France; 5 Centre Paul Papin, Angers, France; 6 Institut de Cancérologie de la Loire, Saint-Etienne, France; 7 Centre Jean Bernard, Le Mans, France; 8 Centre Alexis Vautrin, Vandoeuvre-les-Nancy, France; 9 Centre Léon Bérard, Lyon, France; 10 CHU Croix Rousse, Lyon, France; 11 CHU Kremlin Bicêtre, Paris, France; 12 Institut Bergonié, Bordeaux, France; 13 Institut Gustave Roussy, Villejuif, France

**Keywords:** unknown primary neoplasms, practice guidelines

Carcinomas of unknown primary site are metastatic malignant epithelial tumours whose primary site cannot be identified during pretreatment assessment. They are characterised by their slow local development and their high metastatic potential. The primary site remains unknown in 20–50% of the patients, but the results from autopsies show that the primary tumours are most often located in the pancreas, lung, gut or kidney.

In France, the incidence of carcinomas of unknown primary site is eight out of 100 000 per year, corresponding to between 5 and 7% of the solid tumours in adults. The average age at detection is 60 years old, with slightly more men being affected. The median survival time is only a few months.

The heterogeneity of carcinoma of unknown primary site is due to the different histopathological types and anatomical localisations, making this a difficult topic to cover. In this document, we present the diagnostic strategy based on these two parameters, with the first entry point being the histopathological type. The therapeutic strategies to be used depend on the prognostic factors: specific anatomoclinical entities (neuroendocrine tumours, cervical lymph node metastases from squamous cell carcinoma, axillary lymph node metastases from an adenocarcinoma in women, undifferentiated carcinoma of the mediastinum in young men) and other nonspecific situations. Although primary papillary serous carcinoma is no longer included in the classification of peritoneum carcinomas of unknown primary site, we covered the management in women here in an attempt to be exhaustive.

## OBJECTIVES

The objective was to define guidelines for the management of adult patients with carcinomas of unknown primary site. These guidelines are aimed at health professionals treating these patients with the goal of helping to homogenising clinical practice.

The principal questions addressed in this document are:

- What pathological diagnostic strategies should be used for each localisation?
- To what extent should the primary site be searched for, and what are the limits for this strategy?
- What are the prognostic factors?
- What treatment strategies should be used for each anatomoclinical type?

## METHODS

The details of the full methodology have been previously published (Fervers *et al*, 2001). In summary, a multidisciplinary working group was set up by the French National Federation of Cancer Centres (Fédération Nationale des Centres de Lutte Contre le Cancer–FNCLCC) to review the literature on the management of patients with carcinomas of unknown primary site.

Medline® was searched between 1980 and 2001 using keywords pertinent for each topic covered and this was completed with references provided by the members of the working group. The majority of the articles were in English and French.

After selection and critical appraisal of this literature, the working group defined the ‘Standards’, ‘Options’ and ‘Recommendations’ (SORs) for the management of patients with carcinomas of unknown primary site, based on a synthesis of the best available evidence and expert agreement. These guidelines were then reviewed by a group of independent experts (see the Appendix) and finalised after taking into consideration their comments. SORs are considered as being validated when the members of the working group give their agreement for publication.

When all the members of the working group agree, based on the best available evidence, that a procedure or intervention is beneficial, inappropriate, or harmful, it is classified as a ‘Standard’, and when the majority agree, it is classified as an ‘Option’ (Table 1

**Table 1:** Definition of Standards, Options and Recommendations

Standards	Procedures or treatments that are considered to be of benefit inappropriate or harmful by unanimous decision, based on the best available evidence
	
Options	Procedures or treatments that are considered to be of benefit, inappropriate or harmful by a majority, based on the best available evidence
	
Recommendations	Additional information to enable the available options to be ranked using explicit criteria (e.g. survival, toxicity) with an indication of the level of evidence

). In the SORs, there can be several ‘Options’ for a given clinical situation. ‘Recommendations’ provide additional information that enable the available options to be ranked using explicit criteria (e.g. survival, toxicity) with an indication of the level of evidence. These recommendations thus help clinicians to select an appropriate option. Thus, clinicians can make choices for the management of patients using this information and taking into consideration local circumstances, skills, equipment, resources and/or patient preferences. The adaptation of the SOR to the local situation is allowable if the reason for the choice is sufficiently transparent and this is crucial for successful implementation. Inclusion of patients in clinical trials is an appropriate form of patient management in oncology and is recommended frequently within the SORs, particularly in situations where only weak evidence exists to support a procedure or an intervention.

The type of evidence underlying any ‘Standard’, ‘Option’ or ‘Recommendation’ is indicated using a classification developed by the FNCLCC based on previously published methods. The level of evidence depends not only on the type and quality of the studies reviewed, but also on the concordance of the results (Table 2

**Table 2:** Definition of level of evidence

*Level A*
There exists a high-standard meta-analysis or several high-standard randaomised clinical trials which give consistent results

*Level B*
There exist good quality evidence from randomised trials (B1) or prospective or retrospective studies (B2). The results are consistent when considered together

*Level C*
The methodology of the available studies is weak or their results are not consistent when considered together

*Level D*
Either the scientific data do not exist or there is only a series of cases

*Expert agreement*
The data do not exist for the method concerned, but the experts are unanimous in their judgement

). When no clear scientific evidence exists, judgement is made according to professional experience and consensus of the expert group (‘expert agreement’), and this is then validated by the peer-review process.

This is a translation of the French version of the summary rapport (Bugat *et al*, 2002), which was based on the full-text version in French, available on internet at the following address: http://www.fnclcc.fr. The document will be updated as new evidence becomes available or there is a change in expert agreement.

The list of abbreviations used in this article and their meaning is given in Table 3

**Table 3:** Abbreviations and their meanings

CK	Cytokeratins KL_1_, CK5/6, CK7, CK19, CK20
CD45	Leucocyte differentiation antigens
PS 100	Protein S100
CD20	B lymphocyte
CD3	T lymphocyte
EMA	Epithelial membrane antigen
CD30	Activation antigen
ALK	Anaplastic lymphoma kinase
HMB45, melan A	Melanic markers
SMA	Smooth muscle actin
SMD	Striated muscle desmin
CD31,CD34	Vascular ‘markers’
CD68	Histiocyte ‘marker’
CD99	Primitive neuroectodermal tumour (PNET) ‘marker’
CD117	c-kit protein
VIM	Vimentin
Calretinin, HBME1, WT1	Mesothelial markers
PLAP	Placental alkaline phosphatase
AFP	α-Foetoprotein
βHCG	β-Human chorionic gonadotrophin
CGA	Chromogranin A
SYN	Synaptophysin
NSE	Neurone specific enolase
CEA	Carcinoembryonic antigen
Hep Par1	Hepatocellular antigen
PSA	Prostate specific antigen
TTF1	Thyroid transcription factor 1
GCDFP	Gross cystic disease fluid protein
ER	Oestrogen receptors
PR	Progesterone receptors
GFAP	Glial fibrillary acidic protein
NF	Neurofilaments

.

## PATHOLOGICAL EXAMINATION

### Treatment of samples prior to pathological examination

Samples should be fixed using buffered formalin or AFA (acetic acid, formaldehyde, alcohol) (standard, level of evidence: B2). The standard staining technique is haematoxylin and eosin (standard, level of evidence: B2). Immunohistochemical investigations should be performed using a panel of antibodies (standard, level of evidence: B2). Samples can be frozen directly in liquid nitrogen and then stored in a freezer at −80°C or lower, or stored in liquid nitrogen (option, expert agreement).

### Strategies for specific histopathological types (Figures 1, 2 and 3)

#### 

##### Undifferentiated malignant tumour

**Figure 1:**
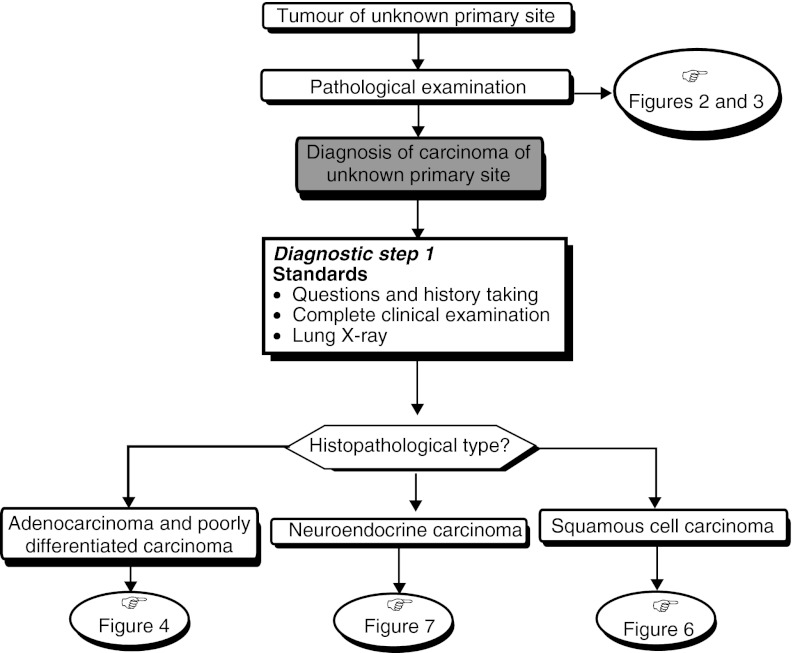
First diagnostic step for carcinoma of unknown primary site.

**Figure 2:**
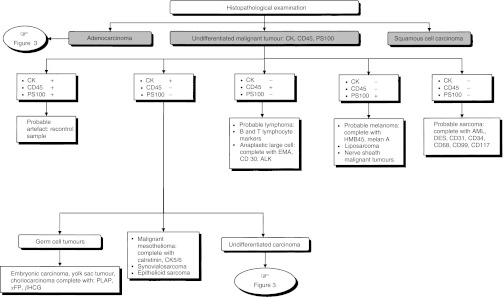
Histopathological diagnosis for carcinoma of unknown primary site.

**Figure 3:**
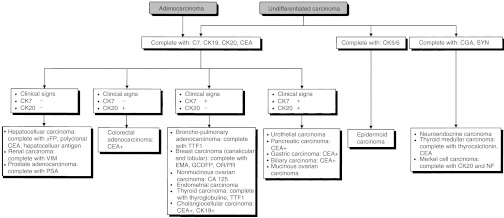
Histopathological diagnosis of undifferentiated carcinoma and adenocarcinoma.

An immunohistochemical investigation should be performed to eliminate the diagnosis of lymphoma, melanoma or germ cell tumour. This should involve the use of a panel of reference antibodies against epithelial antigens (pan-cytokeratins), lymphoid antigens (CD45, CD20, CD3), melanotic antigens (PS100 et HMB45) and germ cell tumour antigens (*α*FP, *β*HCG, PLAP) depending on the clinical presentation (standard, level of evidence: B2).

##### Undifferentiated carcinoma or adenocarcinoma

Neuroendocrine tumour markers should be used in the immunohisto-chemical investigation (e.g. chromogranin, synaptophysin), as well as carcinoma markers (cytokeratins: CK5/6, CK7, CK19, CK20, ACE) and other antibodies depending on the anatomo-clinical presentation (e.g. thyroglobulin, prostate specific antigen (PSA), hormonal receptors) (standard, level of evidence: B2).

### Histopathological examination for carcinoma of unknown primary site

An immunohistochemical investigation for the diagnosis should be performed using an appropriate panel of specific antibodies (standard). This should enable the diagnosis of lymphoma, melanoma, germ cell tumour and sarcoma to be eliminated and the diagnosis of prostate, breast, ovary, thyroid or neuroendocrine tumours to be positively identified. A sample can be frozen to enable typing, cytogenetic and, particularly, molecular biological studies to be performed later (option). The clinician and pathologist should compare their opinions before and after the pathological diagnosis (recommendation, expert agreement).

## DIAGNOSTIC STRATEGY

### Systematic diagnostic assessment

Diagnostic strategy should aim to identify anatomoclinical entities of carcinomas of unknown primary site for which there is a specific treatment (standard, level of evidence: B2). For other anatomoclinical entities, identification of the primary tumour has no impact on the prognostic or therapeutic consequences, thus a systematic complete assessment is unnecessary (standard, level of evidence: B2). The systematic diagnostic assessment is summarised in step 1 in Table 4

**Table 4:** Diagnostic work-up for carcinoma of unknown primary site as a function of their histopathological and anatomic localisation

Step 1	Step 2	Step 3			
Routine diagnostic work-up	Specific work-up depending on the histopathology	Specific work-up as a function of the localisation			
		Localisation	Standards	Options	Recommendations
	**Neuroendocrine carcinoma**		There are no standards	There are no options	There are no recommendations
Carcinoma of unknown primary site	**Adenocarcinoma and undifferentiated carcinoma**	Midline adenopathies	Testicular ultrasound (level of evidence: B2) Chest-abdominal CT scan (level of evidence: B2)	There are no options	There are no recommendations
*Standards*	(Standards, level of evidence: B2):	Mediastinal adenopathies	Testicular ultrasound and chest-abdominal CT scan to eliminate a germ cell tumour in men	There are no options	There are no recommendations
Pathological evaluation (level of evidence: B2)	*For women*	Cervical and/or supraclavicular adenopathies	Testicular ultrasound (in the presence of an associated midline adenopathy) Chest-abdominal CT scan)	There are no options	Panendoscopy and neck and face CT scan, serology for Epstein–Barr virus or detection of DNA by *in situ* hybridisation to eliminate diagnosis of a undifferentiated nasopharyngeal carcinoma (expert agreement)
Clinical history (level of evidence: B2)	Mammography				
Clinical examination (level of evidence: B2)	Ultrasound or pelvic CT scan				
Chest X-ray (level of evidence: B2)	*For men*				
No complete work-up (level of evidence: B2)	Serum PSA, *α*FP, *β*HCG assays				
		Axillary adenopathy (women)	Breast ultrasound^a^ (level of evidence: C)	There are no options	There are no recommendations
			Breast MRI^a^ (level of evidence: C)		
		Inguinal adenopathy	There are no standards	There are no options	There are no recommendations
		Liver metastases	Woman: αFP assay if undifferentiated carcinoma	Coloscopy	Coloscopy in the presence exclusive, resectable liver metastases (expert agreement)
		Lung metastases	Women: *β*HCG assay	There are no options	There are no recommendations
			Men: chest-abdominal CT scan and testicular ultrasound		
		Bone metastases	There are no standards	There are no options	Bone scintigraphy and standard X-rays of painful zones
		Brain metastases	There are no standards	There are no options	There are no recommendations
		Single metastasis	There are no standards	Whole-body CT scan (expert agreement) Bone scintigraphy	There are no recommendations
		Pleural effusion	There are no standards	Chest CT scan (expert agreement)	There are no recommendations
		Peritoneal effusion^b^	There are no standards	Abdominal-pelvic CT scan in women	There are no recommendations
	Squamous cell carcinoma	Cervical adenopathy	Panendoscopy (level of evidence: B2)	There are no options	There are no recommendations
			Head and neck CT scan (level of evidence: B2)		
			Diagnostic bilateral amygdalectomy		
		Supraclavicular or axillary adenopathy	There are no standards	Chest CT scan	There are no recommendations
	No specific work-up for this histopathological type	Inguinal adenopathy	Clinical examination of the external genital organs	Pelvic CT scan or ultrasound	There are no recommendations
			Anuscopy and colposcopy		
		Bone metastases	Complete clinical examination with a head and neck examination (expert agreement)	Panendoscopy	Bone scintigraphy and standard X-rays of painful zones (expert agreement)

a Examination to be performed after a ‘negative’ mammography.

b Although this entity is not included in the classification of ‘carcinoma of unknown primary site’ because the primary tumour is known, the therapeutic implications from its diagnosis lead the working group to include this entity in the current SOR document.

CT=computed tomography; PSA=prostate antigen specific; *α*FP=*α*-foctoproteins; *β*HCG=*β*-human chorionic gonado trophin; MRI=magnetic resonance imaging.

.

### Specific work-up to eliminate diagnosis of extragonadal germ cell tumour

The main differential diagnoses for patients with carcinomas of unknown primary site are extragonadal germ cell tumour and lymphoma, because they are potentially curable. The specific work-up for eliminating the diagnosis of extragonadal germ cell tumour, includes a systematic diagnostic work-up and a specific work-up for adenocarcinomas and undifferentiated carcinomas (standard).

### Diagnostic work-up depending on histopathological and anatomic localisation (Figures 4, 5 and 6)

**Figure 4:**
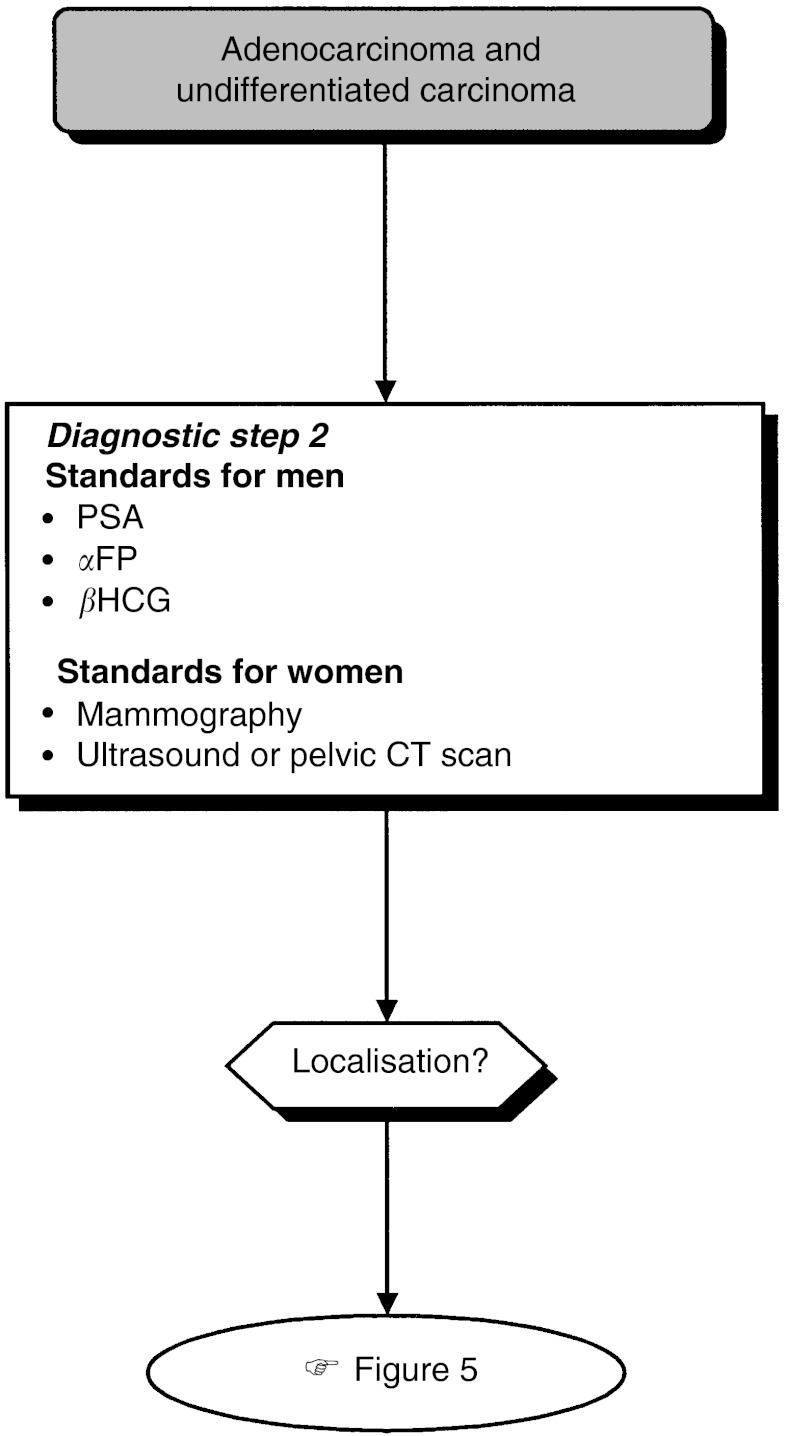
Second diagnostic step for adenocarcinoma and undifferentiated carcinoma.

**Figure 5:**
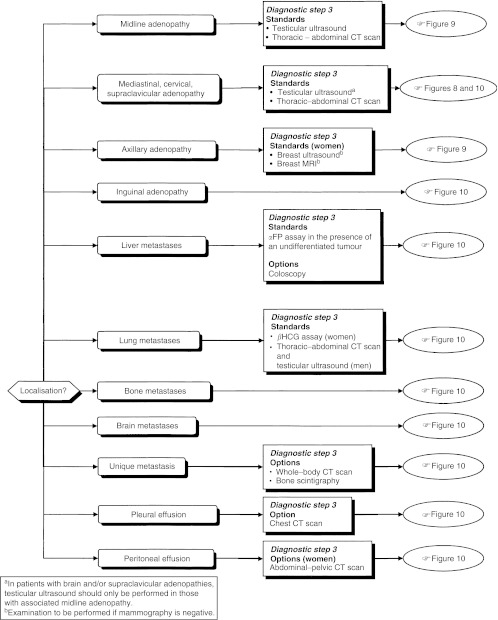
Third diagnostic step for adenocarcinoma and undifferentiated carcinoma.

**Figure 6:**
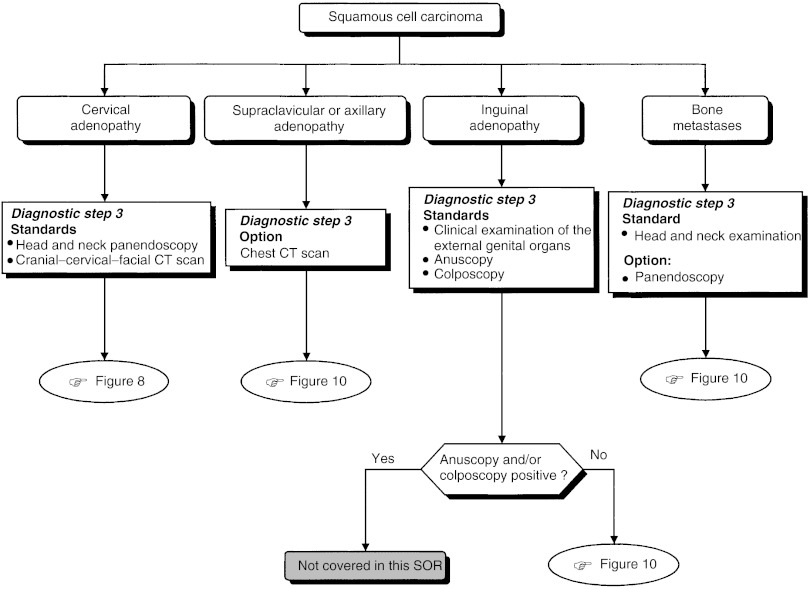
Complementary examinations for squamous cell carcinoma.

The diagnostic steps (steps 2 and 3), performed depending on the histopathological and anatomic localisation, are shown in Table 4.

## PROGNOSTIC FACTORS FOR CARCINOMA OF UNKNOWN PRIMARY SITE

No prospective studies or meta-analyses for prognostic factors have been published, but there are several retrospective studies with coherent results, which suggest that the following are the best prognostic factors: general good health status; women; lymph node metastases; neuroendocrine or squamous cell carcinoma; and few metastatic sites (level of evidence: B2). It is recommended to include patients with carcinomas of unknown primary site in good-quality studies assessing prognostic factors (recommendation).

## TREATMENT STRATEGY

### Treatment of specific anatomoclinical entities

#### 

##### Treatment of neuroendocrine carcinoma (Figure 7)

**Figure 7:**
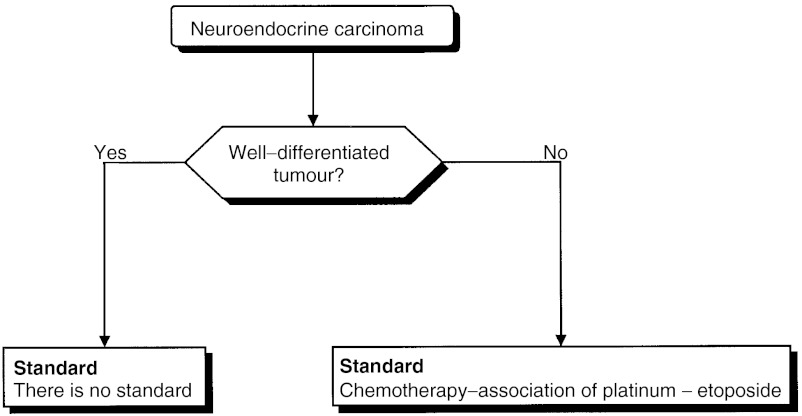
Treatment of neuroendocrine carcinoma.

The treatment of metastases from a neuroendocrine carcinoma is not modified by the identification of the primary site (expert agreement). The management of patients with neuroendocrine carcinoma of unknown primary site should take into consideration the cellular differentiation (standard, expert agreement).

Poorly differentiated forms are considered to be chemosensitive (level of evidence: C). The usual treatment is based on a combination of a platinum salt and etoposide (level of evidence: C). Although the results from clinical trials do not provide evidence for efficacy in terms of increased survival, clinicians should prescribe this treatment (standard, expert agreement). There is no standard for the forms that are well differentiated.

The treatment decision should be based on a multidisciplinary decision taking into consideration the patient's symptoms and the progression of the carcinoma, particularly for those with well-differentiated forms (recommendation).

##### Treatment of cervical lymph node metastases in patients with squamous cell carcinoma (Figure 8)

**Figure 8:**
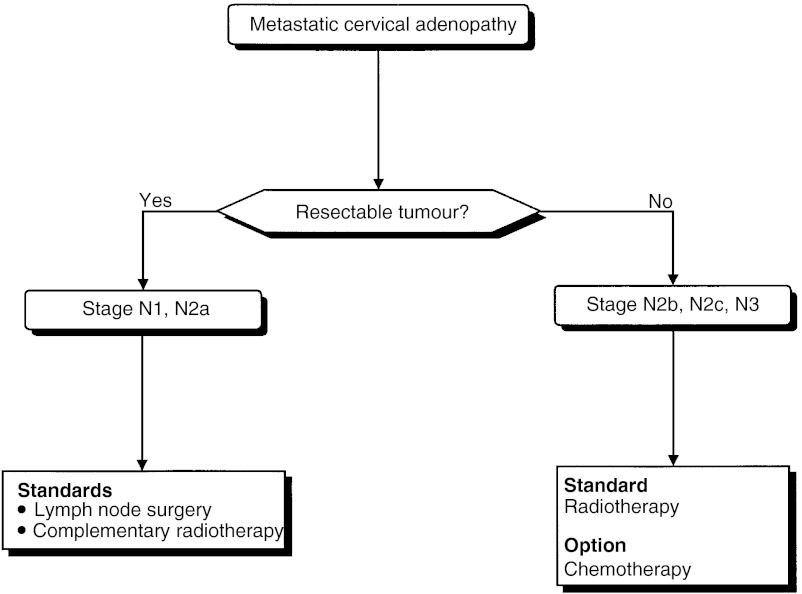
Treatment of cervical lymph node metastases in patients with squamous cell carcinoma.

Patients with cervical lymph node metastases from squamous cell carcinoma should be offered lymph node dissection and complementary radiotherapy (standard, level of evidence: C). If surgery is not possible, radiotherapy should be performed (standard). Chemotherapy can be proposed to patients with tumours that are not suitable for resection or surgery (option).

#### Treatment of axillary lymph node metastases in women with adenocarcinoma (Figure 9)

**Figure 9:**
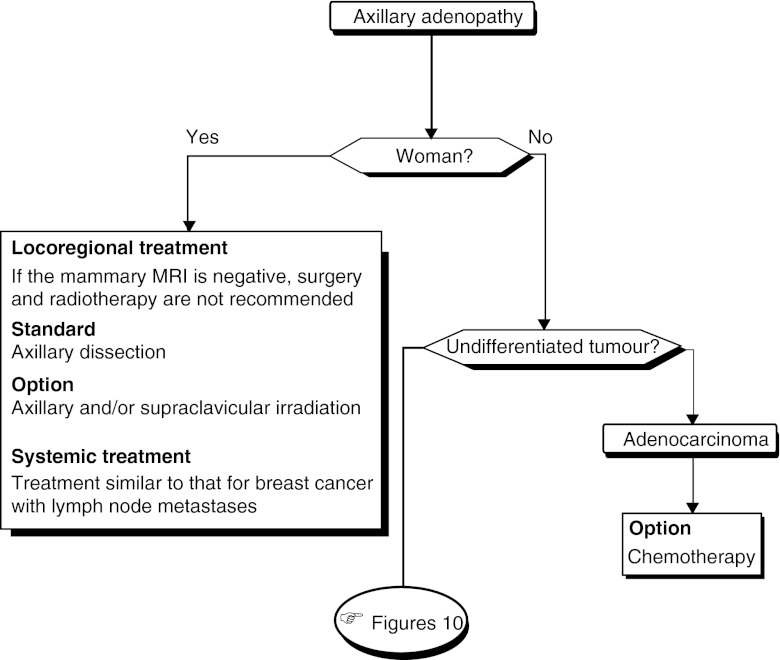
Treatment of axillary lymph node metastases in patients with adenocarcinoma.

Locoregional treatment (breast): If the results from the breast MRI are negative, surgery and breast radiotherapy should not be offered (standard, expert agreement).

Locoregional treatment (axilla): Axillary dissection should be offered (standard, expert agreement). Axillary and/or supraclavicular irradiation can be undertaken (option, expert agreement).

Systemic treatment: The management of these patients should be identical to that for patients with breast cancer with lymph node metastases (recommendation).

##### Treatment of primary papillary serous carcinoma in women

By analogy with ovarian cancer, the standard treatment is tumour reduction by surgery (level of evidence: D) followed by polychemotherapy containing a platinum salt (standard, expert agreement). About six cycles of treatment should be undertaken (recommendation).

#### Treatment for carcinomas of unknown primary site not belonging to a specific anatomoclinical entity (Figure 10)

**Figure 10:**
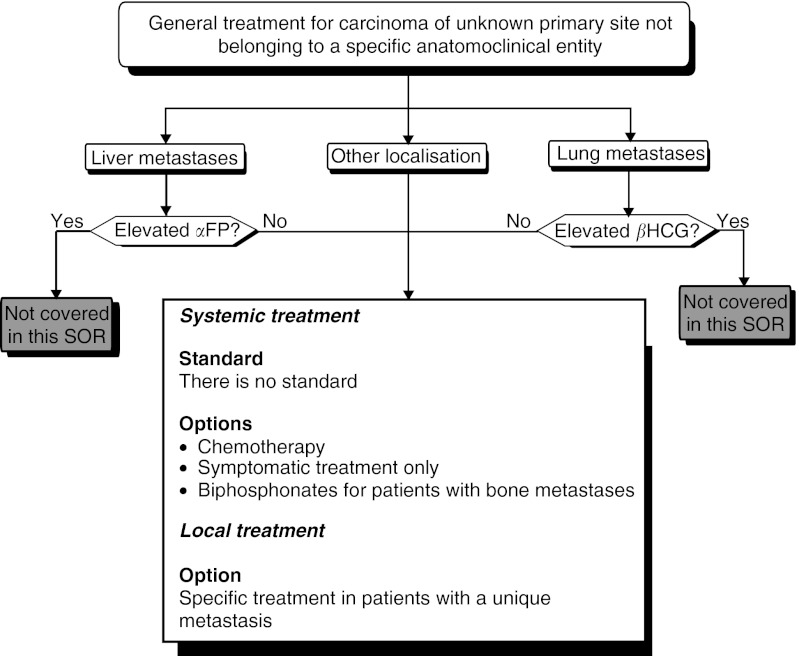
General treatment of carcinoma of unknown primary site not belonging to a specific anatomoclinical entity.

Locoregional treatment: Patients with a single metastatic site can be offered specific treatment (option, expert agreement).

Systemic treatment: Several treatments can be envisaged (options): chemotherapy (level of evidence: B2), symptomatic treatment only, or a treatment based on bisphosphonates in patients with bone metastases. If chemotherapy is prescribed, it is recommended to administer a combination therapy with two drugs, containing cisplatin (recommendation, expert agreement) for patients with a good general health status (WHO performance status of 1 or less). The treatment response should be evaluated early (after two cycles) to avoid treatment in patients with known progressive disease (recommendation).

## Appendix

### Reviewers

C Bailly (Centre Léon Bérard, Lyon, France), A Balaton (Cabinet Médical, Bièvres, France), P Baldet (CHU, Montpellier, France), JF Berdah (CHU, Limoges, France), F Collin (Centre Georges-François Leclerc, Dijon, France), T Conroy (Centre Alexis Vautrin, Nancy, France), M Fabbro (Centre Val d’Aurelle, Montpellier, France), B Fabiani (CHG, Le Mans, France), P Fournel (CHU, Saint-Etienne, France), J Hassoun (Institut Paoli Calmettes, Marseille, France), E Jadaud (CHU, Angers, France), L Laccourreye (CHU, Angers, France), C Louvet (CHU Saint-Antoine, Paris, France), L Malissard (Centre de Radiothérapie et d’Oncologie, La Chaussée-St-Victor, France), JP Metges (CHU, Brest, France), P Morice (Institut Gustave Roussy, Villejuif, France), D Orfeuvre (Centre Hospitalier, Bourg-en Bresse, France), F Penault-Llorca (Centre Jean Perrin, Clermont-Ferrand, France) F Priou (Centre Hospitalier, La Roche sur Yon, France), P Rebattu (Centre Léon Bérard, Lyon, France), S Rémy (Hôpital de la Côte-Basque, Bayonne, France), B Sigal-Zafrani (Institut Curie, Paris, France), I Sillet-Bach (Hôpital, Brive, France), L Tolou (CHG, Rodez, France), P Vielh (Institut Curie, Paris, France), E Voog (Clinique Victor Hugo, Le Mans, France).
